# Comparison of noninvasive continuous arterial blood pressure measured by NICAP with arterial line in elderly patients

**DOI:** 10.1186/s12877-022-02803-3

**Published:** 2022-02-07

**Authors:** Zhao Xu, Hongyang Chen, Hongyu Zhou, Xiaohui Sun, Jun Ren, Hongxia Sun, Chan Chen, Guo Chen

**Affiliations:** 1 Department of Anesthesiology, West China Hospital, Sichuan University, No.37 Guoxue Alley, 610041 Chengdu, China; 2 Department of Anesthesiology, West China Hospital, Sichuan University/ West China School of Nursing, Sichuan University, No.37 Guoxue Alley, 610041 Chengdu, China; 3 Department of Anesthesiology, Xinjiang Production and Construction Corps Hospital, No. 232 Qingnian Road, 830002 Urumqi, China

**Keywords:** Non-invasive, Blood pressure, Monitoring, NICAP, Elderly, Intraoperative

## Abstract

**Background:**

Non-Invasive Continuous Arterial Pressure system (NICAP) allows continuous monitoring, timely detection of hypotension, and avoiding risks from invasive procedures. A previous study showed good comparability of NICAP with arterial line in people with no evidence of cardiovascular disease. Therefore, the goal of this study was to investigate whether NICAP could be accurately applied to elderly patients.

**Methods:**

In this single-centered observational study, forty-one patients above 65 undergoing elective surgeries requiring artery catheterizations were enrolled from July 17, 2020, to June 25, 2021. Radial artery cannulation and NICAP monitoring were started before anesthesia. Blood pressure during the anesthesia induction and the whole surgery, trend of blood pressure changes, time needed for establishing continuous monitoring, and complications were recorded.

**Results:**

A total of 6751 valid pairs of blood pressure measurements were analyzed. In the Bland-Altman analysis, the arithmetic means for systolic, diastolic, and mean arterial pressure were 2.2, 3.3, and 2.8 mmHg, respectively. NICAP and arterial line correlation coefficients for systolic, diastolic, and mean arterial pressure were 0.51, 0.40, and 0.47, respectively. In the trending analysis, the polar concordance rates at 30 degrees were 70.9% for systolic, 67.7% for diastolic, and 69.3% for mean arterial blood pressure. During the anesthesia induction, the arithmetic means for systolic, diastolic, and mean arterial pressure in the Bland-Altman analysis were 1.7, -0.2, and 0.5 mmHg, respectively. NICAP and arterial line correlation coefficients for systolic, diastolic, and mean arterial pressure were 0.72, 0.58 and 0.69, respectively. No severe complications occurred.

**Conclusions:**

NICAP has a poor correlation with the arterial line in elderly patients for the whole surgery or during anesthesia induction. Moreover, it showed poor comparability in the detection of blood pressure change trends with arterial lines. Our findings suggest that NICAP might not be sufficiently accurate to be applied clinically in elderly patients with comorbidities. More accurate calibration and iteration are needed.

## Background

Intraoperative hypotension, whether absolute decrease in mean arterial pressure or hypotensive episodes, is strongly associated with poor postoperative outcomes especially for elderly patients whose vessel elasticity and cardiopulmonary function reserve are significantly reduced due to common comorbidities, such as hypertension and diabetes [1–9]. Early detection of hypotension and individualized maintenance of blood pressure during surgery could reduce risks of postoperative complications and improve the prognosis for elderly patients [1, 9–12].

Current monitoring recommendation of blood pressure taken every 5 min could neglect some hypotension episodes while arterial catheterization as the gold standard could cause severe complications [13, 14]. Non-invasive continuous blood pressure monitoring could be an excellent substitute. Several non-invasive continuous blood pressure monitoring devices with various monitoring mechanisms have been developed such as Nexfin, T-line tensimeter, and Continuous Non-invasive Arterial Pressure (CNAP). Those devices need calibration and correction from cuff blood pressure to form accountable blood pressure readings and took a relatively long time to establish. Non-Invasive Continuous Arterial Pressure system (NICAP) is another monitoring device using two near-infrared light sensors to detect the arterial pulse waveforms of the ear and toe to calculate the pulse wave velocity and formulate arterial blood pressure [15–17]. Its application is simple, quick, and could avoid complications associated with invasive procedures. In addition, due to its mechanism, intermittent calibration from cuff blood pressure is not needed. In a previous validation study, NICAP showed good comparability with invasive blood pressure monitoring and met The Association for the Advancement of Medical Instrumentation (AAMI) standard in people with no evidence of cardiovascular disease [18]. However, the application of NICAP in elderly patients who may have several comorbidities has never been validated before.

Thus, we conducted this observational study to investigate whether NICAP could accurately reflect arterial line blood pressure in elderly patients during surgery. We hypothesized that NICAP was accurate compared with arterial line blood pressure in elderly patients.

## Methods

### Study design

 The Ethics Committee of Clinical and Biomedical Trials, West China Hospital of Sichuan University (No. 2019-136). Written informed consent was obtained from all the participants. The study was conducted in accordance with the Declaration of Helsinki (as revised in 2013). Forty-one patients were enrolled from July 17, 2020, and June 25, 2021. Inclusion criteria were: (1) over the age of 65, (2) undergoing elective surgery in a supine position, (3) requiring arterial catheterization. Patients were excluded if they had: (1) advanced dysfunction of peripheral perfusion, (2) arteriovenous shunts for hemodialysis, (3) vascular surgery of lower extremities, (4) peripheral arterial disease, and (5) anatomical abnormalities or injuries at the NICAP sensor site.

### NICAP monitoring technique

NICAP technology (Zhejiang Union Medical Equipment Co., Ltd.) uses two near-infrared light sensors to detect the arterial pulse waveforms of the ear and toe for calculating the pulse wave velocity (PWV). The system uses instantaneous pattern changes of the pulse waveform to calibrate the PWV. A mathematical model algorithm was established between PWV and blood pressure based on the wave motion equation, elastic mechanics, and hemodynamics [15–17].

### Blood pressure monitoring process

**Fig. 1:**
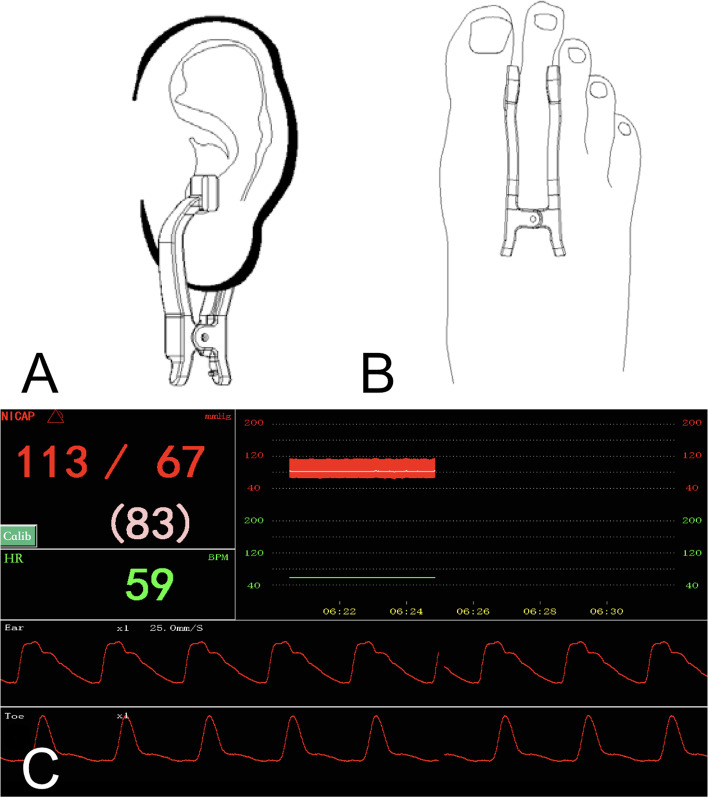
**A** and **B** Application of the ear and toe clamps. **C** Monitor Screen of NICAP showing blood pressure, heart rate, and the pulse waveforms of ear and toe. (Figures cited from the NICAP user manual of Zhejiang Union Medical Equipment Co., Ltd. Witten consent of usage and adaptation was obtained from the company)

Before induction of anesthesia, radial arterial cannulation was performed for all the patients using 20G SURFLO® (TERUMO CHINA HOLDING CO., LTD.) under local anesthesia (2% lidocaine) and arterial blood pressure was monitored (Single Channel Transducer Kit for IBP Monitoring, SCW MEDICATH LTD, Shenzhen, China). Then the NICAP sensors were applied to the patients’ ears and toes according to the manufacturer’s recommendations (Fig. 1). The time needed for cannulation and NICAP monitoring were recorded, respectively. Recordings of systolic (SBP), diastolic (DBP), and mean blood pressure (MAP) from both methods were started at the induction of anesthesia and ended after the patients were transferred to post-anesthesia care unit (PACU) for 1-minute intervals in the anesthesia information system. During induction, blood pressure was recorded every 30 s for 5 min manually. Complications of both methods, including hematoma, hemorrhage, nerve injury, and pressure injury to the skin, were documented.

### Anesthesia procedure

Monitoring of electrocardiography, pulse oximetry, capnography, and body temperature was started when patients arrived at the operating room (OR). After radial artery cannulation and calibration of arterial blood pressure, anesthesia was induced intravenously with midazolam 0.03 mg/kg, sufentanil 0.3ug/kg, propofol 1-2 mg/kg, and cis-atracurium 0.2 mg/kg. After intubation, anesthesia was maintained with 2% sevoflurane or 6% desflurane and remifentanil 0.05-0.2ug/kg/min. All patients were ventilated using a lung-protective strategy including tidal volume of 6-8ml/kg, positive end-expiratory pressure (PEEP) 6-8mmHg, and a frequency to maintain end-tidal carbon dioxide (EtCO_2_) between 35-40mmHg.

The primary outcome of this study was to assess the accuracy of NICAP compared with arterial blood pressure for elderly patients during the whole surgery (excluding the anesthesia induction). Secondary outcomes include blood pressure recorded from the two methods during anesthesia induction, the trend of blood pressure changes of the two methods, the time needed for establishing continuous monitoring for both methods, and complications.

### Statistical analysis

There is no consensus on how many subjects and measurements should be analyzed when performing a repeated measure. Patients enrolled in different studies varied [19–25]. According to the AAMI protocol, validation of new monitoring tools for a specific population requires at least 35 patients. In this study, 41 patients were enrolled. A total of 6751 pairs of data were analyzed. All the data were blinded from the analyzer for statistical analysis. Data were expressed as the mean with standard deviations (SD) or median with the 25th to 75th percentiles (IQR). Categorical variables are shown as numbers (%). Data were tested for normal distribution with the Q-Q plots [26]. Blood pressure differences between methods were analyzed using a dependent t-test for paired measurements. Bland–Altman analysis for multiple measurements per subject was used to assess the bias (mean difference) and precision (SD of the bias) between SBP, DBP, and MAP recorded by the two methods. In addition, correlations between ABP and NICAP were determined by linear regression for the whole repeated paired measurements from all patients. Finally, the polar plots were drawn using SigmaPlot (Systat Software Inc, San Jose, CA) to analyze the two methods’ trending abilities of changes in SBP, DBP, and MAP [27–29]. Concordance rate above 92% was determined to be good, while below 90% was considered to be poor [27]. The Bland-Altman plots and scatter plots were drawn using MedCalc® Statistical Software version 19.7.2 (MedCalc Software Ltd, Ostend, Belgium; https://www.medcalc.org; 2021). Statistical analyses were performed using SPSS (IBM Corp. Released 2019. IBM SPSS Statistics for Macintosh, Version 26.0. Armonk, NY: IBM Corp). Two-sided *p* Values < 0.05 were considered statistically significant.

## Results

A total of 60 potentially eligible patients were screened due to the surgical planning list. Forty-four consented, nine refused to participate, six failed to meet the inclusion criteria, and 1 was canceled due to poorly controlled hypertension. All patients received right radial artery cannulation after Allen’s test was normal. The NICAP waveform recording was determined to be acceptable in 44 patients. NICAP data were missing in 2 patients due to incorrect connection of the device. Radial artery canulation was failed in 1 patient. Finally, data from 41 patients were analyzed. A summary of the baseline characteristics of the patients is presented in Table 1. Total anesthesia time (mean ± SD) was 230.41 ± 98.66 min, and the time of surgery (mean ± SD) was 167.83 ± 86.21 min. Although we obtained 9487 pairs of blood pressure data during the study, 2736 pairs were excluded from the analysis, in which 1523 were due to arterial line flushing artifacts, 385 were due to arterial blood pressure data missing, and 828 due to NICAP data missing in the anesthesia information system. Therefore, 6751 valid pairs of simultaneous arterial (ART) and NICAP measurements were analyzed. The median blood pressure data pairs per patient was 123 (95-232). A total of 410 pairs of blood pressure measurements were obtained during anesthesia induction. 130 pairs from 7 patients were excluded from the analysis due to artifacts or incorrect connection. Thus, a total of 280 valid pairs of data from 34 patients were analyzed. The median blood pressure data pairs per patient during induction was 9 (7.75-10). The blood pressure results of the whole surgery and during anesthesia induction are shown in Tables 2 and 3. The time needed to establish monitoring for blood pressure was significantly shorter for NICAP compared with arterial catheterization. [ART vs. NICAP (min): 9 (7-13) vs. 2 (2-3), *p* < 0.001] There were no major complications that occurred either from arterial line or NICAP.

**Table 1:** Baseline characteristics of the patients

	Population (*n* = 41)
Age (yr)	72.29 ± 5.40
Gender (M/F)	26/15
BMI (kg/m^2^)	23.49 ± 2.96
ASA (2/3)	20/21
Anesthesia time (min)	230.41 ± 98.66
Surgery time (min)	167.83 ± 86.21
Type of surgery, n (%)	
Laparoscopic resection of rectal cancer	10 (24.4%)
Liver resection	6 (14.6%)
Laparoscopic radical gastrectomy	6 (14.6%)
Laparoscopic colonectomy	5 (12.2%)
Radical prostatectomy	4 (9.8%)
Laparoscopic exploration	3 (7.3%)
Pancreaticoduodenectomy	3 (7.3%)
Total thyroidectomy	2 (4.9%)
Simple mastectomy	1 (2.4%)
Transcervical resection of mediastinal mass	1 (2.4%)
Comorbidities, n (%)	
Diabetes	37 (90.2%)
Hypertension	20 (48.8%)
Coronary heart disease	1 (2.4%)
Smoking within 1 year	33 (80.5%)
COPD	37 (90.2%)
Stroke	1 (2.4%)

Values are mean ± standard deviation or numbers (%)

*BMI* Body mass index

*COPD* Chronic obstructive pulmonary disease

**Table 2:** Blood pressure measurements from all patients

*n* = 6751	ART		NICAP		*P*^a^
	Mean	SD	Mean	SD	
SBP	120.14	18.38	117.60	19.08	<0.000
DBP	62.95	10.43	59.72	12.54	<0.000
MAP	83.64	13.14	81.03	13.74	<0.000

^a^ Paired-samples T test

**Table 3:** Blood pressure measurements during anesthesia induction from 34 patients

*n* = 280	ART		NICAP		*P*^a^
	Mean	SD	Mean	SD	
SBP	132.22	28.87	131.36	26.36	0.430
DBP	68.52	14.66	69.08	13.94	0.454
MAP	89.76	18.20	89.81	16.71	0.946

^a^Paired-samples T test

**Fig. 2:**
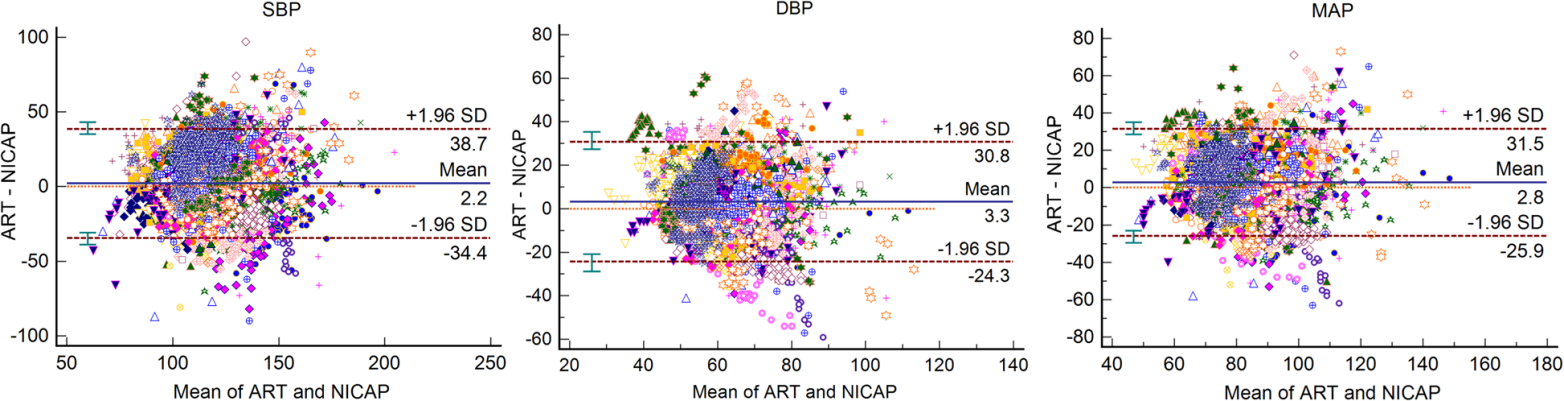
Bland-Altman analysis between NICAP and ART for SBP, DBP, and MAP

**Fig. 3:**
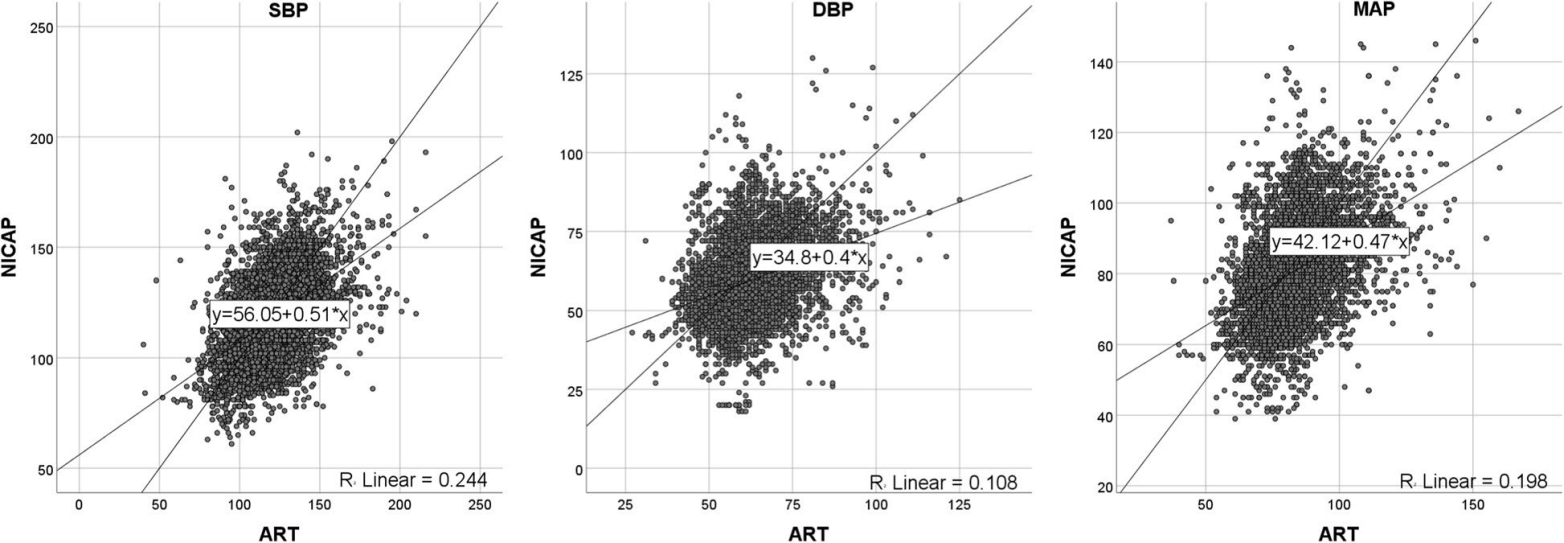
Scatter plots and linear regression results for SBP, DBP, and MAP

**Fig. 4:**
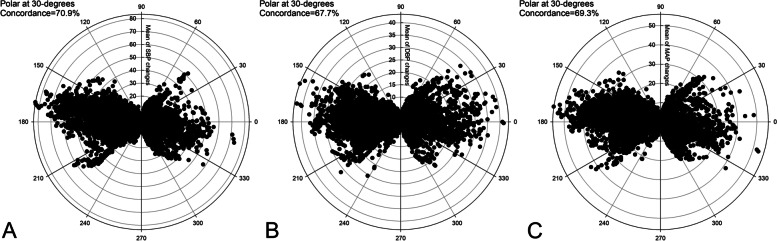
Polar plot analysis on trending ability of changes of systolic (**A**), diastolic (**B**), and mean arterial blood pressure (**C**) measured by NICAP against the arterial line

**Fig. 5:**
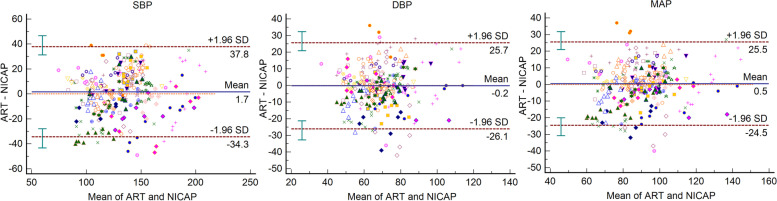
Bland-Altman analysis between NICAP and ART for SBP, DBP and MAP during anesthesia induction

**Fig. 6:**
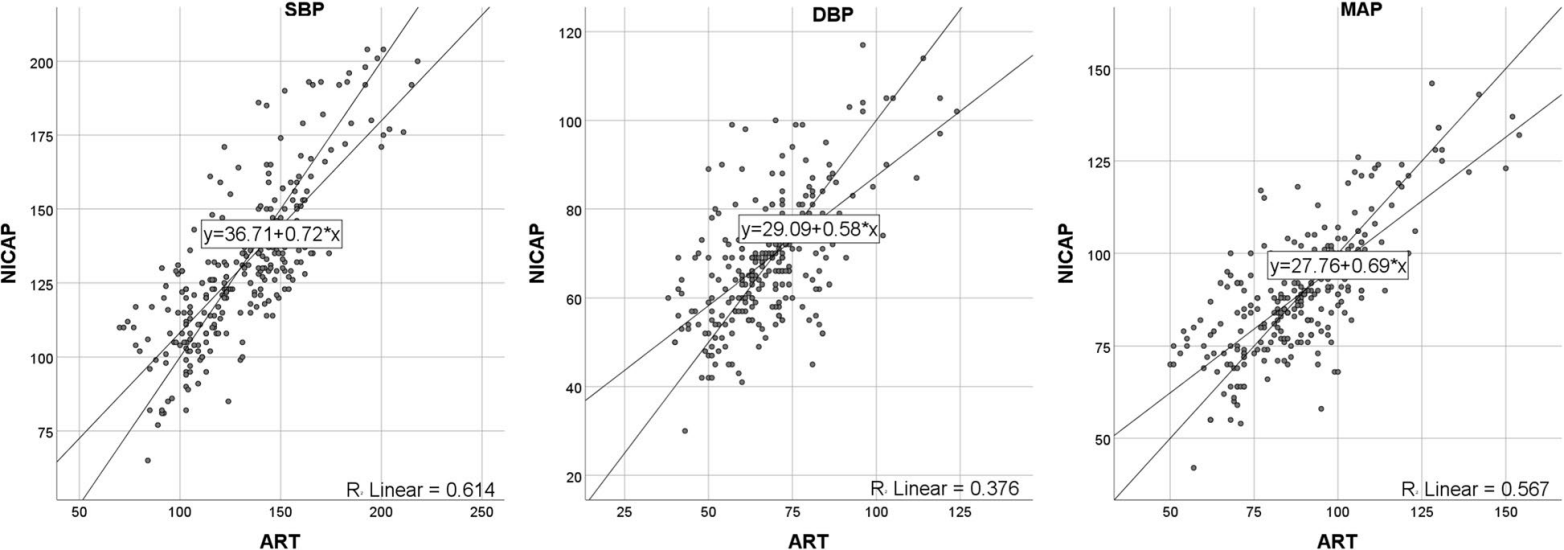
Scatter plots and linear regression results for SBP, DBP, and MAP during anesthesia induction

The results of the Bland-Altman plot for multiple measurements per subject between NICAP and ART are shown in Fig. 2. Bias and precision were 2.19 and 18.86 mmHg in SBP, 3.27 and 13.42 mmHg in DBP, 2.78 and 14.17 mmHg in MAP. Upper and lower limit of agreements (LOAs) were 38.74 and -34.36 mmHg in SBP, 30.84 and -24.31 mmHg in DBP, and 31.46 and -25.90 mmHg in MAP. Although bias fulfilled the AAMI criteria below 5 mmHg, precisions all exceeded the AAMI standard of 8 mmHg. The scatter plots for SBP, DBP, and MAP are shown in Fig. 3. The correlation coefficients were 0.51, 0.40, and 0.47 for SBP, DAP, and MAP, respectively. SBP, DBP, and MAP from NICAP were not strongly correlated with ART. The overall NICAP measurement for elderly patients was lower than ART regardless of SBP, DBP, or MAP. In the polar plot trending analysis, the polar concordance rate at 30 degrees was 70.9% for SBP, 67.7% for DBP, and 69.3% for MAP (Fig. 4). During anesthesia induction, the arithmetic means for systolic, diastolic, and mean arterial pressure in the Bland-Altman analysis were 1.7, -0.2, and -0.5 mmHg, respectively. Bias and precision were 1.72 and 18.31 mmHg in SBP, -0.17 and 12.59 mmHg in DBP, 0.49 and 12.34 mmHg in MAP. Upper and lower LOAs were 37.8 and -34.3 mmHg in SBP, 25.7 and -26.1 mmHg in DBP, and 25.5 and -24.5 mmHg in MAP (Fig. 5). NICAP and arterial line correlation coefficients for systolic, diastolic, and mean arterial pressure were 0.72, 0.58, and 0.69, respectively (Fig. 6). NICAP showed poor comparability with the arterial line during anesthesia induction. Moreover, NICAP showed poor ability to detect blood pressure change trends compared with the arterial line.

## Discussion

Our results demonstrated that blood pressure measured by NICAP showed poor correlation with arterial blood pressure in elderly patients either during the whole surgery or during anesthesia induction. Moreover, NICAP had poor comparability with an arterial line in the detection of blood pressure change trends. There were no major complications that occurred either from the arterial line or NICAP.

Recent studies focusing on non-invasive blood pressure monitoring devices suggest that continuous non-invasive beat-to-beat monitoring of blood pressure can effectively and instantaneously detect hypotension during surgeries compared with traditional cuff blood pressure monitoring that usually occurs 3-5 min intermittently [30–33]. However, these continuous non-invasive monitoring devices use different mechanisms and algorithms and showed great heterogeneity in the accuracy of detecting the actual blood pressure. Multiple studies concluded that these devices could not replace traditional cuff and invasive monitoring techniques [34, 35]. Similar to these studies, we also found that NICAP showed a poor correlation with the arterial blood pressure in elderly patients regardless of SBP, DBP, or MAP during the whole surgery. Several reasons might contribute to its inaccuracy. First, the NICAP system algorithm was formulated using intraoperative monitoring data from adults between 18 and 70. However, the mean age of the studied population was 72.29 years old, with the oldest of 87 years old. Aging is associated with many physiological changes in large and small arteries’ structural and functional properties, including arterial diameter, wall thickness, wall stiffness, and endothelial function [36, 37]. As a consequence of increased arterial stiffness, pulse wave velocity increases, and reflected waves return faster [38, 39]. This was a key element in the algorithm of NICAP. Second, comorbidities were generally common in elderly patients. In this study, hypertension was present in 20 (48.8%) and diabetes 37 (90.2%) out of 41 patients. Hypertension and diabetes could also cause many physiological alterations. For elderly patients with multiple comorbidities, the NICAP algorithm might need updates and iteration before clinical application. Third, the influence of involuntary body movements might be a major reason for the inaccuracy. The NICAP system requires two pulse wave readings from ear and toe clamps to correct blood pressure calculation. These two clamps are relatively far from each other in different body parts and thus could be easily affected due to the anesthesia and surgical settings in the operating room. Although muscle relaxants were administered, procedures like bag-valve ventilation and endotracheal intubation during induction could accidentally affect the ear clamp. Correspondingly, surgical manipulation could also affect the ear and toe clamps during the surgery, causing inaccuracy. Compared to other continuous blood pressure monitoring systems using artery contouring techniques, this incidence might be higher [24]. Because of this particular characteristic of NICAP, appropriate surgery type and sites might be a prerequisite for successful application.

Blood pressure readings from NICAP could easily be affected during induction, as aforementioned. Anesthesia induction is considered the most critical phase in surgery due to the most unstable hemodynamics, especially for elderly patients [10, 40]. Accurate detection of hemodynamic changes during induction could facilitate timely treatment from the anesthesiologists, thus avoiding catastrophic events. Although NICAP could be easily established in a timely manner, the inaccuracy limited its application in this high dependent situation. In our daily practice, the arterial line was seldomly established before induction due to pain stimulation while cuff blood pressure monitoring takes relatively longer per test. The application of NICAP during induction could have been an excellent alternative. However, further research, development, and calibration are still needed before it could be applied to elderly patients in situations like this.

NICAP also showed poor comparability with the arterial line in the detection of blood pressure change trends during the surgery. Worthy of note, in the present study, the concordance rate of above 90% was considered has good trending ability. However, this standard was adopted from studies focusing on cardiac output devices [27, 28]. To our knowledge, there was no consensus for good trending ability of blood pressure monitoring devices. Although NICAP has poor trending ability according to this standard, it is still considered by the authors as a good device for anesthesiologists to monitor the blood pressure change trend continuously. It could facilitate timely reactions of anesthesiologists to treat potential hyper- or hypotension episodes before it occurs. Another advantage we found in this study was that NICAP took significantly less time than the arterial line to establish accountable continuous blood pressure readings. We encountered one patient in the PACU that was complicated with postoperative bleeding in the study period. The patient was hypotensive and in a hemorrhagic shock. Traditional cuff blood pressure readings could not be obtained due to low circulating volume, and the arterial line was challenging to establish due to hypotension. In this situation, the NICAP acquired accountable continuous blood pressure readings in a short time. Although arterial line could not be replaced in many clinical cases, timely establishment of continuous blood pressure readings by NICAP could guide the resuscitation and rescue of critical patients giving that the trend of blood pressure changes could be accurately reflected by NICAP. This needs to be further studied in the future.

No complications such as hematoma, pressure injury was recorded in the study. The clamps are light-weighted, and the force applied to the ear and toe is relatively small. This might facilitate prolonged monitoring of non-invasive continuous blood pressure by NICAP in a non-high dependency situation for those high-risk patients. However, the mean surgical time in this study was less than 2 h and could not be interpreted as a prolonged period. Future studies focusing on prolonged monitoring and potential complications should be conducted before a conclusion is drawn.

There were several limitations of this study. Firstly, despite 6751 pairs of valid data analyzed, the sample size of this study was small. Future studies with more subjects are needed. Secondly, the NICAP has many restrictions, and these restrictions may affect its clinical application. Thus, the patients included in this study might not represent all the surgical types. The results of this study should be interpreted with caution. Finally, the mean surgical time of this study was 167.83 min which was less than 3 h. Longer duration might cause some complications such as pressure injury by ear and toe clamps of NICAP. Future studies are warranted to investigate these issues.

## Conclusions

In summary, this study has demonstrated that NICAP has a poor correlation with the arterial lines in elderly patients either during the whole surgery or during anesthesia induction. Moreover, it showed poor comparability with the arterial line in the detection of blood pressure change trends. Our findings suggest that NICAP might not be sufficiently accurate to be applied clinically in elderly patients. More precise calibration and iteration of NICAP are needed for the elderly population.

## Data Availability

The datasets used and/or analyzed during the current study are available from the corresponding author on reasonable request.

## Abbreviations

NICAP: Non-Invasive Continuous Arterial Pressure system
CNAP: Continuous Non-invasive Arterial Pressure
AAMI: The Association for the Advancement of Medical Instrumentation
PWV: Pulse wave velocity
SBP: Systolic blood pressure
DBP: Diastolic blood pressure
MAP: Mean blood pressure
PACU: Post-anesthesia care unit
OR: Operating room
PEEP: Positive end-expiratory pressure
EtCO2: End-Tidal Carbon Dioxide
SD: Standard deviations
IQR: 25th to 75th percentiles
LOAs: Limit of agreements
