# Correction to: Comparison of noninvasive continuous arterial blood pressure measured by NICAP with arterial line in elderly patients

**DOI:** 10.1186/s12877-022-02835-9

**Published:** 2022-02-28

**Authors:** Zhao Xu, Hongyang Chen, Hongyu Zhou, Xiaohui Sun, Jun Ren, Hongxia Sun, Chan Chen, Guo Chen

**Affiliations:** 1 Department of Anesthesiology, West China Hospital, Sichuan University, No.37 Guoxue Alley, Chengdu, 610041 China; 2 Department of Anesthesiology, West China Hospital, Sichuan University/ West China School of Nursing, Sichuan University, No.37 Guoxue Alley, Chengdu, 610041 China; 3 Department of Anesthesiology, Xinjiang Production and Construction Corps Hospital, No. 232 Qingnian Road, Urumqi, 830002 China

**Correction to: BMC Geriatr (2022) 22:108**

**https://doi.org/10.1186/s12877-022-02803-3**

After publication of this article [1], the authors reported that the abstract contains 6 incorrect values. The original article [1] has been updated.
